# Clinical Applications, Opportunities, and Implementation Challenges of AI in Emergency Medicine: A Narrative Review

**DOI:** 10.7759/cureus.114581

**Published:** 2026-08-15

**Authors:** Nabeel Ashiq, Fouzia Munir, Adil Yousaf

**Affiliations:** 1 Emergency Medicine, Hamad Medical Corporation, Doha, QAT; 2 Pharmacology, Green Health Pharmaceutical Company, Riyadh, SAU

**Keywords:** artificial intelligence, clinical decision support, digital health, emergency department, emergency medicine, large language models, machine learning, triage

## Abstract

EDs operate in high-acuity, time-sensitive, and resource-constrained environments where crowding, diagnostic uncertainty, boarding, variable patient acuity, and increasing documentation demands place substantial pressure on clinicians and health systems. AI, including machine learning, deep learning, natural language processing, computer vision, predictive analytics, and large language models (LLMs), is increasingly being investigated as a supportive technology for emergency care. This narrative review summarizes current and emerging applications of AI in emergency medicine, with emphasis on triage and risk stratification, diagnostic decision support, emergency imaging, patient-flow optimization, LLMs, and implementation governance. The strongest near-term applications are likely to be those that address clearly defined clinical or operational tasks, use available electronic health record or imaging data, integrate into existing workflows, and undergo prospective validation in the intended setting. AI may support earlier recognition of high-risk patients, prioritization of critical imaging findings, prediction of hospital admission or deterioration, drafting and summarization of clinical documentation, and operational planning. However, evidence remains uneven across use cases. Important barriers include limited external validation, dataset shift, bias, false alarms, alert fatigue, lack of interpretability, privacy risks, uncertain liability, regulatory complexity, and difficulty demonstrating patient-centered outcome improvement. LLMs introduce additional risks, including hallucination, outdated information, unsafe recommendations, and privacy concerns, and should not be used as autonomous decision-makers in emergency care. Responsible implementation requires human oversight, local validation, post-deployment monitoring, equity assessment, governance, and alignment with emergency clinician workflows. AI should be viewed as a tool to augment, not replace, emergency physicians and multidisciplinary ED teams.

## Introduction and background

EDs are complex clinical environments where clinicians must make rapid decisions under conditions of uncertainty, variable acuity, crowding, and limited resources. ED crowding is associated with delays in assessment and treatment, increased boarding, staff workload, and poorer patient and system-level outcomes [1,2]. These pressures are particularly important because emergency medicine often functions as the front door of the hospital and the safety net for unscheduled care.

Diagnostic uncertainty is an inherent feature of emergency medicine. Patients may present early in the course of illness, with incomplete histories, nonspecific symptoms, limited prior records, language barriers, intoxication, altered mental status, or multiple comorbidities. A systematic review supported by the Agency for Healthcare Research and Quality highlighted that diagnostic errors in the ED are clinically important, although estimates vary because of methodological limitations and case definitions [3].

AI, including machine learning, deep learning, natural language processing, computer vision, predictive analytics, and large language models (LLMs), is increasingly being investigated in emergency care (Table 1). Contemporary reviews have described applications across triage, risk prediction, imaging, sepsis detection, clinical decision support, documentation, and operations [4-7]. Emergency medicine is a high-impact environment for AI because ED decisions are frequent, time-sensitive, data-rich, and closely linked to downstream hospital resource use. However, the potential value of AI should not be overstated. Technical performance in retrospective datasets does not guarantee improved patient outcomes, clinician acceptance, or safe deployment. AI tools may fail when transported across institutions, may worsen inequities if trained on biased data, and may create new safety problems through automation bias, alert fatigue, or workflow disruption [8,9].

**Table 1:** Major AI technologies and their potential applications in emergency medicine. LLM, large language model

AI technology	Example application	Potential benefit	Key limitation
Machine learning	Prediction of hospitalization, deterioration, sepsis, or intensive care need at triage	May identify high-risk patients earlier and support resource allocation	Requires high-quality data, external validation, calibration monitoring, and workflow integration
Deep learning	Image, ECG, waveform, and high-dimensional clinical data interpretation	Can detect complex patterns that are difficult to encode manually	Often less interpretable and may be sensitive to dataset shift
Natural language processing	Extraction or summarization of triage notes, physician notes, and discharge narratives	Can convert unstructured ED text into useful structured information	Performance depends on documentation quality, terminology, and local language patterns
Computer vision	Detection or prioritization of critical imaging findings	May shorten time to review for selected urgent findings	May fail with poor image quality, artifacts, or populations not represented in training data
Predictive analytics	Forecasting crowding, waiting time, admission, or bed demand	May support operational planning and bed management	Highly dependent on local workflows and hospital capacity
LLMs	Drafting discharge instructions, summarizing notes, and supporting education	May reduce documentation burden and improve communication support	Risk of hallucination, privacy breaches, bias, and unsafe advice without review
Clinical decision support	Alerts, risk scores, and prioritization flags integrated into the electronic health record	May support earlier recognition and consistent action	Can cause alert fatigue, automation bias, and medicolegal concerns

## Review

Overview of AI in emergency medicine

AI is an umbrella term for computational methods designed to perform tasks that usually require human intelligence. In healthcare, AI frequently refers to systems that classify images, predict risk, extract meaning from clinical text, generate language, detect patterns in physiologic signals, or support decision-making. Emergency medicine uses data streams that are particularly suitable for AI research, including triage information, vital signs, nursing notes, physician documentation, laboratory results, ECGs, imaging, medication records, prior utilization, and disposition outcomes [4-7].

Machine learning methods learn statistical relationships from data and can be used to predict outcomes such as admission, deterioration, return visits, sepsis, or intensive care need. Deep learning, a subset of machine learning based on multilayer neural networks, is often used for high-dimensional data such as imaging, ECGs, waveforms, or large collections of clinical variables. Natural language processing converts free text into structured information or summaries, which is relevant because much ED information is contained in unstructured notes [4-7,9-12].

Computer vision refers to algorithms that interpret visual data, including radiographs, CT, ultrasound images, photographs, or video. LLMs are generative AI systems trained on large bodies of text and capable of producing summaries, explanations, drafts, translations, and responses to prompts. Clinical decision support systems may incorporate one or more of these technologies and deliver outputs such as alerts, risk scores, prioritization flags, or suggested next steps. In emergency medicine, such systems should be designed to support clinician judgment rather than replace clinical responsibility [13,14].

AI for ED Triage and Risk Stratification

Triage is one of the most widely studied emergency medicine AI use cases. ED triage must rapidly assign acuity and identify patients at risk for deterioration, hospitalization, critical care, or time-sensitive intervention. Large retrospective studies have shown that machine learning models can predict clinical outcomes from triage data in adult and pediatric ED populations, including hospitalization and critical illness outcomes [9,10]. These findings support the concept that routinely collected triage information may contain signals that can improve risk stratification, although retrospective performance is not equivalent to clinical benefit.

AI-assisted triage may combine age, sex, chief complaint, vital signs, comorbidities, medication history, previous utilization, presenting narrative, early laboratory results, and electronic health record-derived features. Potential benefits include faster prioritization, earlier recognition of critical illness, improved resource allocation, and more consistent identification of high-risk presentations. Sepsis prediction has been a major focus, but results remain mixed. External validation of the Epic Sepsis Model found substantially lower performance than expected, highlighting the importance of independent validation and calibration in the local population [11]. A prospective multisite study of a machine learning early warning system for sepsis reported improved outcomes after implementation, but such results require careful interpretation in relation to workflow, clinician response, and local implementation factors [12].

AI has also been explored for chest pain and cardiac risk stratification, including ECG-based and prehospital approaches to acute coronary syndrome [13]. Stroke and trauma prioritization may involve prediction of large-vessel occlusion, intracranial hemorrhage, or injury severity using imaging, vital signs, mechanism of injury, and clinical variables [14,15]. Despite these opportunities, AI-assisted triage carries important risks. False positives can increase alert fatigue and resource use, while false negatives can create false reassurance. Bias may arise if training data reflect historical under-triage of certain populations. Any triage AI must therefore undergo prospective evaluation, equity assessment, and workflow testing before routine use [13,14].

AI in Diagnostic Decision Support

Diagnostic decision support in emergency medicine aims to help clinicians generate, prioritize, or test differential diagnoses using multimodal data such as symptoms, vital signs, laboratory results, ECGs, imaging, medication history, previous encounters, and clinical documentation. Potential applications include chest pain, sepsis, pulmonary embolism, stroke, abdominal pain, toxicology, trauma, and undifferentiated presentations. These tools may be particularly attractive for supporting junior clinicians, reminding teams of uncommon but dangerous diagnoses, and prompting reassessment when new data change the risk profile [4-7].

The appeal of diagnostic AI is clear: emergency clinicians must decide quickly while balancing common benign presentations against rare catastrophic diagnoses. AI may help identify overlooked patterns, prompt missing workup, or support differential diagnosis generation. However, diagnostic support systems are difficult to validate because ED diagnoses evolve over time, reference standards may be imperfect, and many patients are discharged without definitive diagnoses. Models may perform well on curated test cases but less well in real ED practice, where data are incomplete, noisy, and time-dependent [4-7].

The main risks are overreliance, automation bias, incorrect recommendations, poor interpretability, and failure to account for evolving clinical information. Automation bias is particularly important when clinicians accept AI suggestions despite conflicting clinical evidence. Recent discussion of assistive AI in clinical decision support emphasizes that automation bias can cause harm when clinicians defer to algorithmic recommendations that appear authoritative but are wrong or poorly contextualized [8]. For this reason, diagnostic AI should present uncertainty, explain key inputs where possible, and be implemented as advisory support rather than as an autonomous diagnostic authority.

AI in Emergency Imaging and Point-of-Care Diagnostics

Emergency imaging is among the most mature areas of healthcare AI because images are data-rich, standardized, and linked to discrete findings. AI has been studied for CT-based detection or prioritization of intracranial hemorrhage, ischemic stroke, pulmonary embolism, cervical spine injury, and trauma-related findings. A systematic review of AI for ischemic stroke and large-vessel occlusion described the potential role of automated imaging analysis, while also emphasizing the need for validation and careful integration into clinical pathways [14]. Deep learning algorithms have also been evaluated for detection of critical head CT findings, illustrating the potential for AI to prioritize studies that require urgent review [15].

X-ray applications include fracture detection, pneumothorax, pneumonia, pulmonary edema, misplaced lines and tubes, and other chest abnormalities. In principle, AI can support image triage, reduce time to radiologist review for critical findings, and assist clinicians in settings with limited immediate specialist availability. Point-of-care ultrasound AI remains an emerging area, with possible uses in acquisition guidance, view classification, and interpretation support. In ED practice, however, ultrasound interpretation is highly operator-dependent and context-dependent, so prospective validation and workflow evaluation remain essential before broad clinical adoption [4-7].

Imaging AI must be interpreted in clinical context. Image quality, scanner differences, acquisition protocols, patient positioning, pediatric versus adult populations, artifacts, prevalence shifts, and site-specific workflows can all affect performance. An algorithm trained on high-quality datasets may perform differently in a crowded ED where studies are incomplete, motion-degraded, or obtained under resuscitation conditions. Imaging AI should complement radiology and emergency physician interpretation, not replace the responsible clinician [4-7,15,16].

An additional consideration for safe implementation of imaging AI is the reporting of calibrated confidence and predictive uncertainty. A model’s predicted probability does not necessarily reflect the reliability of its prediction, particularly when images are ambiguous or differ from the data used during model development. Uncertainty quantification can provide information about the reliability of individual predictions, while calibration helps determine whether predicted probabilities correspond to observed clinical outcomes [17-19]. In emergency radiology, this distinction is important because AI outputs may be used to prioritize potentially time-critical examinations or support diagnostic decisions. An apparently confident but unreliable prediction could result in inappropriate prioritization or false reassurance, whereas identification of high-uncertainty cases could prompt closer review by a radiologist or emergency clinician. Therefore, imaging AI systems should, where feasible, communicate calibrated confidence or uncertainty alongside their predictions rather than presenting outputs as definitive findings. Such reporting may improve the interpretability and safe use of AI-assisted radiological prioritization and decision support, although standardized approaches for evaluating and communicating uncertainty remain an area of ongoing research [17-19].

AI in ED Operations and Patient Flow

Operational AI aims to improve ED throughput and hospital coordination. Common applications include prediction of ED crowding, waiting time estimation, bed management, staffing optimization, ambulance diversion risk, hospital admission prediction, discharge planning, and resource allocation. ED crowding is a multifactorial problem that reflects input, throughput, and output constraints; therefore, AI tools that only predict crowding without enabling operational action may have limited effect [1,2].

Admission prediction models may use demographic information, chief complaint, triage acuity, vital signs, laboratory orders, prior utilization, medication lists, and early clinician documentation. Machine learning models have been developed to predict hospital admission at ED triage, suggesting that early prediction could help bed managers, inpatient teams, and ED leaders anticipate demand [16]. Related tools may estimate waiting times, identify patients likely to need advanced imaging, or forecast staffing needs based on historical patterns and real-time arrivals.

Operational AI is highly local. A model trained in one hospital may not perform well in another because staffing structures, inpatient capacity, referral pathways, observation units, diagnostic protocols, and discharge practices differ. Models may also degrade when hospital processes change. Implementation requires clear operational ownership, integration with bed management workflows, continuous monitoring, and evaluation of outcomes that matter to patients and staff, such as waiting time, length of stay, boarding, left-without-being-seen rates, safety events, and clinician workload [16].

LLMs in emergency medicine

LLMs have attracted substantial interest because emergency medicine relies heavily on text: triage notes, physician documentation, nursing narratives, discharge instructions, handoffs, radiology reports, laboratory interpretation, and patient communication. A 2024 scoping review described possible roles for LLMs in emergency medicine, including documentation support, education, patient communication, and clinical decision support, while emphasizing that evidence remains early and implementation risks are substantial [20].

Potential ED applications include drafting or summarizing notes, generating discharge instructions at appropriate literacy levels, translating patient-facing instructions, extracting key information from prior records, summarizing long clinical notes, supporting handoffs, preparing patient education materials, assisting literature review, and creating simulation or teaching cases. Some retrospective work has suggested that GPT-4 may perform well on diagnostic tasks using ED cases, but such studies are not equivalent to prospective clinical validation and should not be interpreted as proof of safe autonomous use [21]. A 2026 Science study evaluated a model on physician reasoning tasks, adding to evidence that LLMs can perform complex clinical reasoning tasks under experimental conditions while also raising questions about how such performance translates into real clinical care [22].

LLMs pose specific risks. They can hallucinate, omit important negatives, generate plausible but incorrect explanations, produce outdated recommendations, mishandle uncertainty, amplify bias, and expose sensitive patient data if not deployed in secure environments. Emergency care is especially vulnerable because decisions are time-sensitive and often made with incomplete information. LLMs should therefore not be used as autonomous decision-makers in emergency care. Safe use requires institutional governance, human review, privacy safeguards, audit trails, evaluation against local clinical scenarios, and clear boundaries around patient-facing and clinician-facing outputs [20-23].

Ethical, legal, and regulatory considerations

Ethical AI implementation in emergency medicine must prioritize patient safety, equity, privacy, transparency, and accountability. WHO has identified ethical challenges and risks in health AI and has articulated principles intended to ensure that AI benefits patients and health systems without undermining autonomy, safety, transparency, or equity [23]. WHO has also issued regulatory considerations for AI in health, emphasizing lifecycle oversight, risk management, transparency, and post-deployment monitoring [24].

Algorithmic bias is a central concern. Models trained on historical data may reproduce disparities related to race, ethnicity, sex, age, disability, insurance status, language, socioeconomic position, geography, or access to care. In emergency medicine, such bias could affect triage priority, pain management, imaging decisions, sepsis alerts, or disposition recommendations. Bias mitigation requires representative development datasets, subgroup performance evaluation, fairness monitoring, and governance mechanisms that include clinicians and patient safety leaders [23,24].

Data privacy and security are particularly important because ED records contain sensitive, high-volume, and often unstructured information. AI systems should comply with applicable privacy law, institutional policy, cybersecurity requirements, and data-use agreements. Liability remains uncertain when AI contributes to clinical decisions. Emergency clinicians should retain responsibility for patient care, but institutions and vendors also have duties related to validation, monitoring, training, usability, and incident response. Regulatory considerations are evolving, including approaches to software as a medical device, predetermined change control plans for AI-enabled device software functions, and good machine learning practice principles [8,24].

Implementation challenges in real-world EDs

Successful AI implementation depends less on model accuracy alone and more on whether the tool improves a real clinical or operational process. ED workflows are fast, interruption-prone, and multidisciplinary. A tool that requires extra clicks, produces poorly timed alerts, or lacks a clear action pathway may increase workload rather than improve care. Human-centered design and collaboration with emergency physicians, nurses, advanced practice clinicians, radiologists, pharmacists, informaticians, administrators, and patient safety teams are essential [8,24].

Integration with electronic health records is a recurring barrier. AI outputs must be delivered at the right time, to the right clinician, in a format that supports action. Triage alerts may need to appear before rooming; imaging alerts must integrate with radiology worklists; sepsis alerts require clear escalation pathways; and operational predictions must be visible to flow coordinators and bed managers. Alert fatigue is a major risk if AI outputs are frequent, nonspecific, or poorly calibrated [25,26].

Implementation also requires training, maintenance, interoperability, governance, and budget planning. Models may require recalibration or retirement when patient populations, documentation practices, laboratory assays, imaging protocols, or hospital workflows change. Evaluation should measure clinical outcomes, process outcomes, safety, equity, usability, and cost, rather than only area under the receiver operating characteristic curve or other technical metrics. Reporting frameworks such as CONSORT-AI and DECIDE-AI may support more transparent evaluation of AI interventions and early-stage clinical decision support systems [25,26].

Opportunities and future directions

Future emergency medicine AI is likely to move toward multimodal systems that combine clinical notes, vital signs, laboratory results, ECGs, imaging, medications, prior records, and operational data. Such systems could support real-time risk prediction, personalized emergency care, and dynamic reassessment as new information becomes available. The most useful systems will likely be those that solve specific clinical or operational problems and are evaluated prospectively in the setting where they will be used.

AI-assisted disaster response and mass casualty triage may eventually support resource allocation, patient tracking, surge prediction, and communication, but many civilian ED applications remain future-facing rather than established. AI-supported tele-emergency medicine may also help remote clinicians access decision support, translation, specialist input, or documentation assistance, but privacy, connectivity, liability, and local workflow constraints must be addressed before broad adoption [8,23,24,27].

Safer LLMs may support documentation, patient education, translation, simulation, and literature synthesis, but they must be evaluated under realistic emergency medicine conditions. Future research should prioritize prospective trials, pragmatic implementation studies, external validation, subgroup performance, model-impact evaluation, and governance frameworks. The field also needs clearer standards for auditability, monitoring, updating, incident reporting, and retirement of AI tools when they no longer perform safely.

## Conclusions

AI has substantial potential to support emergency medicine, particularly in triage, risk stratification, imaging prioritization, sepsis and deterioration prediction, documentation support, and operational optimization. The strongest current applications are those that address clearly defined problems, use available clinical or imaging data, and can be integrated into existing workflows without increasing cognitive burden. Even in these areas, AI should be treated as a supportive technology rather than an autonomous clinical authority.

Clinical adoption should be cautious, evidence-based, and governed by transparent institutional policies. Human oversight, local validation, post-deployment monitoring, equity assessment, privacy protection, workflow integration, and regulatory compliance are essential. The goal should not be to replace emergency physicians or multidisciplinary ED teams but to augment clinical judgment, improve situational awareness, reduce preventable delays, and support safer, more efficient emergency care. As a narrative review, this study is subject to the limitations of a non-systematic search strategy and potential selection bias. Furthermore, given the rapidly evolving nature of AI research, the findings should be interpreted as a contemporary synthesis of the available evidence.
